# Effects of General and Sexual Aggression on the Job, Health and Psychological Outcomes of Women Reservists in the U.S. Armed Forces

**DOI:** 10.3390/bs16030393

**Published:** 2026-03-09

**Authors:** Armando X. Estrada, Wendi L. Benson, Jawaria A. Abbasi

**Affiliations:** 1 Department of Policy, Organizational & Leadership Studies, Temple University, Philadelphia, PA 19122, USA; 2 Department of Psychology, Marshall University, Huntington, WV 25755, USA

**Keywords:** workplace aggression, psychological well-being, women in the military

## Abstract

We examined the unique and joint effects of general and sexual aggression on the job, health, and psychological outcomes of women in the reserve component of the U.S. military with varying activation and deployment experiences (*n* = 13,541). We expected that general and sexual aggression would negatively influence women’s job, health, and psychological outcomes, and that the effects of general aggression would be stronger than the effects of sexual aggression on these outcomes. Further, we evaluated whether aggressive behaviors combined in an additive, adaptive or amplified manner to influence women’s outcomes. Consistent with our hypotheses, both general and sexual aggression experiences were associated with decreased satisfaction with work, coworkers and leaders, lower organizational commitment, poorer physical health and increased psychological distress; the effects of general aggression were stronger than the effects of sexual aggression on women’s outcomes; and the combined effects of general and sexual aggression on women’s outcomes were best characterized in terms of an adaptive response. Results were consistent for women reservists regardless of their activation or deployment experience. We discuss various implications of our findings for future research in this area.

## 1. Introduction

Aggression in the workplace is a serious problem, with wide-ranging consequences for individuals and their organizations (Hershcovis & Barling, 2010; Lapierre et al., 2005). Data from national surveys suggest that nearly 40% of U.S. employees experience aggressive behaviors at work (Alfandari et al., 2022; Schat et al., 2006; Workplace Bullying Institute, 2010). Media portrayals of physical violence and death highlight the importance of this workplace problem, but research shows that less egregious acts are more common (Keashly & Harvey, 2005; Schat et al., 2006). For example, 41% of U.S. employees experienced psychological forms of aggression, whereas only 4% experienced physical violence in the 12 months preceding the survey (Schat et al., 2006), and 73% of women working in private industry reported experiencing trauma from nonfatal workplace violence in 2020 (U.S. Bureau of Labor Statistics, 2024). Other research has documented the negative effects of aggression on an employee’s job, psychological, and physical health outcomes (Chan et al., 2008; Lapierre et al., 2005; Pacheco et al., 2021).

This research has advanced our understanding of the nature, extent, and impact of aggressive behaviors on employees’ personal and professional lives (Chan et al., 2008; Hershcovis & Barling, 2010; Lapierre et al., 2005). However, several limitations exist in the literature. First, most studies examine aggressive behaviors in isolation (Brinker et al., 2023; Hershcovis & Barling, 2010; Lapierre et al., 2005), ignoring important linkages among different forms of aggression in the workplace (e.g., Yragui et al., 2017; Raver & Nishii, 2010). Secondly, research suggests that general and sexual aggression appear to have distinct patterns of effects that are theoretically meaningful and empirically distinguishable from one another (Schnittker, 2022; Hershcovis & Barling, 2010; Lapierre et al., 2005). Finally, research to date has used small samples of convenience involving full-time employees within civilian organizations. Thus, there is a need for studies examining multiple forms of aggressive behaviors and outcomes with large probability-based samples of employees with varying employment characteristics.

This paper begins to address these limitations by examining the impact of both general and sexual aggression on job, health and psychological outcomes of women reservists in the U.S. military with varying activation and deployment experiences. The data include responses from women reservists who have not been previously activated in the past 24 months and were not currently activated or deployed (i.e., *Traditional Reservists*); women who have been activated in the past 24 months and were not currently activated or deployed (i.e., *Reservist with Previous Activation History*); women who have been activated in the past 24 months, are currently activated, but are not currently deployed (*Reservist with Recent Activation History*); and women who have been activated in the past 24 months and were currently activated and deployed (*Reservist with Recent Activation and Deployment History*). These data are used to examine the unique effects of general and sexual aggression on women’s satisfaction with work, coworkers, and leaders, and women’s psychological and physical health outcomes. Additionally, these data are used to evaluate competing hypotheses involving the effects of both general and sexual aggression on women’s outcomes. Before presenting the findings of this study, a review of relevant theory and research on aggressive behaviors and outcomes is presented to contextualize the hypotheses under investigation.

## 2. Literature Review

### 2.1. The Unique Effects of General and Sexual Aggression on Employee Outcomes

*General Aggression*. Workplace aggression involves “efforts by individuals to harm others with whom they work or have worked, or the organizations in which they are presently or were previously employed” (R. A. Baron & Neuman, 1996, p. 395). Aggressive behaviors may include verbal and physical behaviors that can be characterized on a passive–active continuum and can be overt or covert (Neuman & Baron, 1998). While cases of physical violence or death receive significant media attention, most instances of workplace aggression among employees involve “expressions of hostility” (Avtgis & Chory, 2010; R. A. Baron & Neuman, 1998). Such hostility is typically verbal or symbolic in nature and may include rumors, verbal assaults, or offensive language (LeBlanc & Barling, 2004) as well as general forms of workplace incivility and bullying (Cortina et al., 2001; Einarsen, 1999). Importantly, research indicates that experiencing general aggression can lower morale, job satisfaction, and commitment (Caillier, 2021; LeBlanc & Kelloway, 2002), decrease psychological well-being (Hogh et al., 2005; LeBlanc & Kelloway, 2002), and result in poorer physical health (Khubchandani & Price, 2015; Frone, 2000). Accordingly, we hypothesize the following:

**H1.**  *General aggression will negatively influence women’s ratings of satisfaction with work, coworkers, and leaders as well as their ratings of organizational commitment, general health, and psychological distress.*

*Sexual Aggression.* Sexual aggression involves a pattern of sexually coercive or non-consensual behavior in which individuals attempt to engage in sexual activity with another person without their given consent (Farris et al., 2008). Most often, sexual aggression in the workplace involves sexually harassing behaviors (Hershcovis & Barling, 2010; Lapierre et al., 2005). Research indicates that anywhere from 15% to over 70% of women experience some kind of sexually harassing behavior while at school or work (Hill & Silva, 2005; Illies et al., 2003). Like general aggression, the experience of sexual harassment can lower job satisfaction and organizational commitment (Willness et al., 2007), interfere with interpersonal relations at work (Gutek & Bikson, 1985; Willness et al., 2007), decrease psychological well-being (Chan et al., 2008; C. Howard et al., 2024; Willness et al., 2007), and physical health (Siuta & Bergman, 2019; Krieger et al., 2005). Accordingly, we hypothesize the following:

**H2.**  *Sexual aggression will negatively influence women’s ratings of satisfaction with work, coworkers and leaders as well as their ratings of organizational commitment, general health and psychological distress.*

### 2.2. Differential Effects of General and Sexual Aggression on Employee Outcomes

Meta-analyses by Lapierre et al. (2005), as well as Hershcovis and Barling (2010), indicate that the effects of general aggression appear to be stronger than the effects of sexual aggression on employees’ job, health, and psychological outcomes. Lapierre et al. (2005) found that general aggression had a stronger negative effect than sexual aggression on individual ratings of job satisfaction. Hershcovis and Barling (2010) found that the negative effects of general aggression on satisfaction with work, coworkers, and supervisors were stronger than the negative effects of sexual aggression on these outcomes, and that the negative effects of general aggression on affective commitment, job stress, and psychological well-being were stronger than the negative effects of sexual aggression on these outcomes. Accordingly, we hypothesize the following:

**H3.**  *The effects of general aggression on women’s ratings of satisfaction with work, coworkers and leaders, as well as their ratings of organizational commitment, general health and psychological distress, will be stronger than the effects of sexual aggression on these outcomes.*

### 2.3. The Joint Effects of General and Sexual Aggression on Employee Outcomes

While meta-analytic findings suggest that general and sexual aggression may differentially influence outcomes, it is worth noting that findings from these studies concern the main effects of aggression on employee outcomes but not their joint effects. Several theories have been used to explain how multiple types of stressors may combine to influence individual outcomes. Life stress theory suggests that, while certain stressors may impact people in different ways, the cumulative effect of all stressors combines to produce an additive effect on a person’s life (Holmes & Rahe, 1967; Rahe & Arthur, 1978). Thus, a person experiencing multiple types of stressors (e.g., general and sexual aggression) may suffer an additional negative effect every time a different stressor is experienced. Adaptation-level theory (Helson, 1964) suggests that multiple factors influence people’s responses to a given stimulus, e.g., a stressor. Individuals evaluate stimuli against internal norms to produce a behavioral response, and these responses can change as new stimuli are presented, as the effects of old stimuli diminish, or as internal norms change. Accordingly, an adaptive effect implies that, while multiple stressors may independently influence a person’s behavioral responses, their combination would not be significantly worse because the person learns to adapt their behavioral responses due to their exposure to other forms of aggressive behaviors (e.g., sexual aggression or general aggression). Cognitive–energetical theory (Hockey, 1997) suggests that responses to stressors are governed by the strategic allocation and control of energy resources required to produce a behavioral response. Accordingly, behavioral responses to a given stressor will affect subsequent allocation and control of energy resources to other stressors, such that it amplifies a person’s behavioral response to subsequent stressors. Thus, an amplification effect implies that the experience of multiple forms of aggression may combine to magnify each other and result in worse outcomes for individuals.

Raver and Nishii (2010) evaluated competing hypotheses involving the joint effects of multiple types of aggression on job, health, and psychological outcomes of samples of university employees and employees drawn from the Study Response Project database of U.S. adults.^1^ They found no support for the amplification effect and suggested that the most parsimonious way to understand how multiple forms of aggression combined to influence women’s outcomes was in terms of an adaptive response. Of particular importance for our purposes, they found that experiencing high levels of general aggression led to consistently low commitment and job satisfaction regardless of one’s level of ethnic or sexual aggression. A slightly different pattern of results was observed for the effects of ethnic, sexual and generalized aggression on employees’ psychological and health outcomes. Specifically, only generalized aggression emerged as a significant predictor of life satisfaction and physical health ratings when entered simultaneously with ethnic and sexual aggression, but experiencing ethnic and sexual aggression accounted for 1% of additional variance when compared with experiencing generalized aggression alone. Both general and ethnic aggression ratings were predictive of anxiety and depression, and all three forms of aggression predicted additional variance when compared with experiencing generalized aggression alone. Thus, they concluded that for the health and psychological outcomes, a slight additive effect best characterized the way in which multiple forms of aggression may combine to affect these outcomes. Taken together, these findings suggest that while general and sexual aggression may independently influence employees’ outcomes, their combination would not be significantly worse on employees’ outcomes than the effects of experiencing one type of aggression alone.

While Raver and Nishii (2010) were among the first to propose and evaluate competing hypotheses involving the joint effects of multiple forms of aggression on an individual’s job, health and psychological outcomes, there are several limitations that warrant caution in the interpretation of their findings. Although their studies employed a rigorous design that used psychometrically sound measures of multiple forms of aggression and outcomes, their samples were small^2^ and the results were not consistent across the two studies they conducted. Given the highly selective nature of the research samples used in the study, coupled with the inconsistent findings across their studies, it is reasonable to question whether these data were robust enough to adequately test the proposed hypotheses. Accordingly, based on the theoretical rationale and empirical findings from Raver and Nishii (2010), we proposed to evaluate the following hypotheses:

**H4a.**  *Experiencing multiple forms of aggression will combine in an additive manner such that the addition of a second form of aggression will predict significant variance in a person’s outcomes beyond that experienced by one type alone.*

**H4b.**  *Experiencing multiple forms of aggression will combine in an adaptive manner such that the experience of general aggression and sexual aggression in conjunction will not predict significant incremental variance in a person’s outcomes when compared with one type alone.*

**H4c.**  *Experiencing multiple forms of aggression will combine in an amplified manner such that the experience of general aggression and sexual aggression in conjunction will predict significant incremental variance in a person’s outcomes when compared with one type alone.*

### 2.4. Summary and Justification for the Present Study

We examine hypotheses involving the unique and joint effects of general and sexual aggression on the job, health and psychological outcomes of women reservists with varying activation and deployment experiences in the U.S. military. These data offer a unique opportunity to examine the effects of these types of aggression on women’s jobs, health and psychological outcomes in a population that is unparalleled in terms of its structure and function.

First, the U.S. Armed Forces have a unique type of organizational culture (Dunivin, 1994; Elron et al., 1999; Redmond et al., 2015), which is characterized by the organized use of legitimized aggression (Janowitz, 1971; Koeszegi et al., 2014); a professional ethos that places high regard on discipline, obedience, courage, trust and self-sacrifice and emphasizes the primacy of the group over the individual (Collins, 1998; Hillen, 1999); and a masculine-warrior image that identifies and extols military service in terms of masculine norms (Dunivin, 1994; Pendlebury, 2018). These organizational characteristics may uniquely predispose military organizations and their members to both experience and enact aggressive behaviors at work. As such, the data from the present study offer a unique opportunity to evaluate hypotheses involving the unique and joint effects of these forms of aggression on outcomes for a distinct type of organization.

Secondly, the military reserves are a unique type of military organization within the U.S. Armed Forces. Reserve component personnel usually have civilian roles that are more central to their lives than their military roles (J. E. Edwards, 2001; J. L. Howard, 2006). Reserve component personnel reside in cities and communities around the United States and may not necessarily be located near a military installation like their active-duty counterparts (J. L. Howard, 2006). Moreover, until the recent Global War on Terror, reserve component personnel did not typically interact with one another on a continual basis like their active-duty counterparts (i.e., 24 h a day/7 day a week). Unlike their active duty counterparts, who live and work in military installations, they are in constant contact with members of their organizations on a more continual basis (i.e., 24 h/7 days per week) in both military installations and, while deployed to geographic regions worldwide, interactions among members of the military reserve vary significantly based on their activation status, as well as their deployment status. Collectively, these qualities of the reserve component can directly impact the nature, extent and outcomes associated with the experience of aggression among women within the reserve component of the U.S. Armed Forces. Indeed, while reserve component personnel may have served on active duty, it was not uncommon for this experience to be limited to initial training and indoctrination received upon entrance into the military—i.e., basic training, and military occupational training received after initial training—i.e., advanced training (J. L. Howard, 2006). Given that the reserve component differs in meaningful ways from the active component, it is important to study the impact of aggression and its outcomes in military organizations that vary significantly in terms of their structure, function and quality of their members’ interactions. For these reasons, these data provide a unique opportunity to test the proposed hypotheses with a large probability-based sample of women reservists who have diverse employment characteristics within the U.S. Armed Forces.

Thus, based on the aforementioned theory, empirical research, and organizational characteristics, we expect that general and sexual aggression will negatively predict women’s job, health and psychological outcomes (Hypothesis 1 and 2), and that the effects of general aggression on women’s job, health and psychological outcomes will be stronger than the effects of sexual aggression on these outcomes (Hypothesis 3). Finally, we evaluate competing hypotheses involving the joint effects of general and sexual aggression on women’s job, health and psychological outcomes—namely, that aggressive behaviors may combine in an additive, adaptive and amplified manner to influence women’s outcomes (Hypotheses 4a, 4b, and 4c).

## 3. Method

### 3.1. Participants and Procedures

Data from the “2004 Sexual Harassment Survey of Reserve Component Members” (Lipari et al., 2005) was used to evaluate the hypotheses under investigation. Questionnaire packets were mailed to a stratified random sample of 76,031 reserve component members of the U.S. Armed Forces between March and June of 2004. The packets included a cover letter describing the general purpose of the study and confidentiality safeguards and included multi-item scales described below. Three waves of postal follow-up reminders and three waves of email follow-up mailings were sent to individuals who had not responded every two weeks after the initial questionnaire packets were sent. A total of 26, 443 eligible members returned usable surveys (men = 12,902, 49%; women = 13.541, 51%), yielding an adjusted weighted response rate of 42%. Because the present study focuses on women’s aggression experiences and outcomes, we describe analyses of data from the 13,541 female respondents included in this study.

Examination of the demographic characteristics indicated that a majority of the respondents were between the ages of 30 and 34 years. Approximately 13% of the respondents had a high school diploma, 51.7% completed some college, and 35.3% had completed a 4-year degree or graduate training. A total of 57% of the respondents were single, and 43.5% were married. A total of 39% of the respondents were junior enlisted rank (e.g., E1–E4); 44% were senior enlisted rank (e.g., E5–E9); 0.8% were warrant officers (e.g., W1–W5); 6.6% were junior grade officers (e.g., O1–O3); and 10% were senior grade officers (e.g., O4–O6). Respondents included members from each of the Military Departments (56.2% Army Reserve/National Guard, 13% Naval Reserve, 3.6% Marine Corps Reserve, and 23.6% Air Force Reserve/National Guard).

### 3.2. Measures

The “2004 Sexual Harassment Survey of Reserve Component Members” included several multi-item scales assessing background and workplace information, satisfaction and retention intentions, health and well-being, aggression-related experiences, and attitudes toward readiness and personnel policies and practices (Lipari et al., 2005). Because the primary purpose of this paper was to examine the relationship between general and sexual aggression on women’s job, health, and psychological outcomes, we describe only those items used in our analyses.

*General Aggression.* Respondent’s experiences of general aggression were measured with 10 items adapted from Glomb and Liao (2003) for use in the Department of Defense (Defense Manpower Data Center, 2005; Lipari et al., 2005). Items assessed the frequency with which a respondent experienced aggressive behaviors at work. Instructions asked respondents to indicate “How often during the past 12 months have you been in a military workplace situation where military personnel, civilian employees, and/or DoD contractors have targeted you with any of the following behaviors?” (e.g., saying offensive or crude things about you; insulting or criticizing you; flaunting status or power over you), using a 5-point response scale ranging from *never* (1) to *very often* (5). Scale scores were computed by averaging across items, with higher scores indicating greater levels of *general aggression*. Cronbach’s alpha coefficient for the scale was 0.94.

*Sexual Aggression.* Respondent’s experiences of sexual aggression were measured with the Sexual Experiences Questionnaire developed for the U.S. Department of Defense (SEQ-DoD; Fitzgerald et al., 1999; Stark et al., 2002). The SEQ-DoD contains multiple items assessing respondents’ experiences of four general categories of unwanted sex related behaviors, including *sexist behaviors* (e.g., “put you down or was condescending to you because of your sex?”), *crude or offensive behaviors* (e.g., “repeatedly told sexual stories or jokes that were offensive to you?”), *unwanted sexual attention* (e.g., “made unwanted attempts to stroke, fondle or kiss you?”) and *sexual coercion* (e.g., “treated you badly for refusing to have sex?”). Instructions asked respondents whether they had experienced any unwanted sex related behaviors from male coworkers or supervisors in the previous 12 months using a 5-point response scale ranging from *never* (1) to *very often* (5). Scale scores were computed by averaging across items, with higher scores indicating greater levels of *sexual aggression*. Cronbach’s alpha coefficient for the scale was 0.93.

*Satisfaction with Work, Coworkers and Leaders.* Eight items taken from J. E. Edwards et al. (1997) were used to assess respondents’ satisfaction with *work* (e.g., “You like the kind of work you do”) and *coworkers* (e.g., “The people in your workgroup tend to get along”), and four items were used to assess respondents’ satisfaction with *leaders* (e.g., “You are impressed with the quality of leadership in your military workgroup”). All items were presented in Likert-type format with a scale ranging from *strongly disagree* (1) to *strongly agree* (5). Scale scores were computed by averaging across items within each scale, with higher scores indicating greater satisfaction with *work*, *coworkers* and *leaders*. Cronbach alpha coefficients were 0.90 for the *work satisfaction* scale, 0.88 for the *coworker satisfaction* scale, and 0.78 for the *leader satisfaction* scale.

*Affective Commitment.* Four items from Gade et al. (2003) were used to assess respondents’ *affective commitment* to the reserve component (e.g., “I feel a strong sense of belonging to my reserve component”). Items were presented in Likert-type format with a scale ranging from *strongly disagree* (1) to *strongly agree* (5). Scores were computed by averaging across items, with higher scores indicating greater *affective commitment* to the reserve component. Cronbach’s alpha coefficient for the scale was 0.92.

*Physical and Psychological Outcomes.* Four items taken from the general health perceptions subscale of the Short-Form Health Survey (SF-36; Ware & Sherbourne, 1992) were used to assess respondents’ *general physical health* (e.g., “I am as healthy as anybody I know”). Items were presented in Likert-type format with a scale ranging from *definitely false* (1) to *definitely true* (4). Scale scores were computed by averaging across items, with higher scores indicating better *physical health*. Cronbach’s alpha coefficients for the scale was 0.79. Ten items taken from the Perceived Stress Scale (PSS, Cohen & Williamson, 1988) were used to assess respondents’ *psychological distress* (e.g., “felt nervous and stressed”). Instructions asked respondents to indicate “How many times over the past month they had perceived stress in their lives using a 5-point response scale ranging from *never* (1) to *very often* (5).” Scale scores were computed by averaging across items, with higher scores indicating higher levels of *psychological distress*. Cronbach’s alpha coefficient for the scale was 0.88.

*Demographic Questionnaire.* Items assessing participants’ gender, level of education, race/ethnicity, marital status, rank, and branch of service were included as part of a demographic questionnaire. Participants selected their gender (e.g., [1] male; [2] female), level of education (e.g., [1] less than 12 years of school to [9] advanced professional degree), race/ethnicity (e.g., Hispanic, non-Hispanic White, Black or African American, American Indian or Alaska Native, Asian, Native Hawaiian or Pacific Islander, or some other race), marital status (e.g., married, never married), rank (e.g., E1 to O6), and branch of service (e.g., Army, Navy Marines and Air Force) from a list of categories for each variable respectively.

## 4. Results

Data analyses proceeded in several steps. A series of correlation and hierarchical linear regression analyses were performed to evaluate Hypotheses 1, 2 and 3—i.e., unique and differential effects of sexual and general aggression on women’s rating of satisfaction, commitment, and physical and psychological health. And moderated regression analyses were performed to evaluate the joint effects of general and sexual aggression on women’s outcomes—i.e., Hypothesis 4a, *additive effect*, Hypothesis 4b, *adaptive effect*, and Hypothesis 4c, *amplified effect*. A Bonferroni correction was employed to hold the experiment-wide alpha level at 0.01. Analyses were performed separately for each of the four groups, including *Traditional Reservists*—i.e., women reservists who had not been previously activated in the past 24 months and were not currently activated or deployed; *Reservist with Previous Activation History*—i.e., women who had been activated in the past 24 months and were not currently activated or deployed; *Reservist with Recent Activation History*—i.e., women who had been activated in the past 24 months and were currently activated but not deployed; and *Reservist with Recent Activation and Deployment History*—i.e., women who had been activated in the past 24 months and were currently activated and deployed. Table 1 and Table 2 display correlation coefficients for all study variables for each of the four groups of reservists included in the study.

### 4.1. Hypotheses 1 and 2: Unique Effects of General and Sexual Aggression on Outcomes

Hierarchical linear regression analyses were performed to evaluate Hypotheses 1 and 2—independent effects of general and sexual aggression on women’s outcomes. For these analyses, we entered demographic characteristics in the first step, and sexual and general aggression scores in the second step for each outcome across each of the four groups of reservists in our study. Table 3 summarizes the results of these analyses. As expected, results of regression analyses of general and sexual aggression scores on women’s ratings of satisfaction, commitment, general health and psychological distress were in the predicted direction and statistically significant, with effect sizes ranging from 0.02 to 0.29 (see Table 3).

As predicted by Hypothesis 1, general aggression scores were predictive of women’s ratings of satisfaction with work, coworkers, and leaders; affective commitment; and general health and psychological distress among all groups of reservists. As predicted by Hypothesis 2, sexual aggression scores were largely predictive of women’s rating of satisfaction with work, coworkers, and leaders; affective commitment; and general health and psychological distress among all groups of reservists. The only exceptions to this general conclusion involved the relationship between sexual aggression and coworker satisfaction among reservists with recent activation history, which was not statistically significant, and the relationship between sexual aggression and coworker satisfaction, general health and psychological distress among reservists with recent activation and deployment history, which were not statistically significant. Nonetheless, it is worth noting that the magnitudes of the effect of general and sexual aggression on women’s outcomes were remarkably consistent across each of the four groups of reservists. Thus, these results provide empirical support for the independent effects of general and sexual aggression on women’s job, health and psychological outcomes predicted in Hypotheses 1 and 2.

### 4.2. Hypothesis 3: Differential Effects of General and Sexual Aggression on Outcomes

Next, we performed a series of hierarchical linear regression analyses to evaluate Hypothesis 3—differential effects of general and sexual aggression on women’s outcomes. For these analyses, we ran two regression models. In the first model, we entered demographic variables in the first step, followed by sexual aggression scores in the second step and general aggression scores in the third step for each of the outcome variables for all groups of reservists in our study. In the second model, we entered demographic variables in the first step, followed by general aggression scores in the second step and sexual aggression scores in the third step for each outcome variable for all groups of reservists in our study. This strategy allowed us to examine how the order of entry would influence the magnitude of the effects associated with general and sexual aggression on women’s outcomes.

As expected, results of regression analyses involving the differential effects of general and sexual aggression on women’s ratings of satisfaction with work, coworkers, and leaders, as well as their ratings of organizational commitment, general health, and psychological distress, were in the predicted direction and statistically significant, with effect sizes ranging from 0.01 to 0.20 (see Table 4). As predicted by Hypothesis 3, the effects of general aggression on women’s ratings of satisfaction with coworkers and leaders and their ratings of organizational commitment were stronger than the effects of sexual aggression on these outcomes for all groups of reservists, regardless of how the predictors were entered into the models (see Table 4). A slightly different pattern of results, though still consistent with Hypothesis 3, was observed for the effects of general aggression on women’s ratings of satisfaction with work when compared to the effects of sexual aggression on this. Specifically, we found that the effects of sexual aggression were stronger than the effects of general aggression when we entered sexual aggression scores before entering general aggression scores (effect sizes ranging from 0.01 to 0.07). However, the magnitude of these effects changed when the order of entry of these variables was reversed—i.e., the effects of general aggression were stronger (effect sizes ranged from 0.03 to 0.08) than the effects of sexual aggression on work satisfaction (effect sizes ranged from 0.01 to 0.02). This pattern of findings was consistent across all groups of reservists (see Table 4). Examination of zero-order correlations reveals small differences in the relationship between general aggression and work satisfaction and sexual aggression and work satisfaction—correlations differ by 0.01–0.02 in absolute value, and the absolute values were stronger for general than for sexual aggression (see Table 1 and Table 2). Taken together, these findings suggest that while general and sexual aggression may have differential impact on women’s ratings of satisfaction with coworkers, leaders and their ratings of organizational commitment, the experience of general and sexual aggression may have comparable effects on women’s ratings of satisfaction with work.

A similar pattern of results was also observed with regard to the effects of general aggression on women’s ratings of general health and psychological distress when compared to the effects of sexual aggression on these outcomes. Specifically, we found that the effects of sexual aggression were stronger than the effects of general aggression when we entered sexual aggression scores before entering general aggression scores (effect sizes ranged from 0.01 to 0.10). However, the magnitude of these effects changed when the order of entry of these variables was reversed—i.e., the effects of general aggression (effect sizes ranged from 0.02 to 0.14) were stronger than the effects of sexual aggression on work satisfaction (effect sizes ranged from 0.00 to 0.01). The pattern of findings was consistent for all groups of reservists with the exception of reservists with recent activation and deployment history (see Table 4). Examination of zero-order correlations reveals small differences in the relationships among general aggression, general health and psychological distress, and among sexual aggression, general health and psychological distress—correlations differ 0.01–0.10 in absolute value, and the absolute values are stronger for general than for sexual aggression (see Table 1 and Table 2). Taken together, these findings suggest that the effects of general aggression on women’s ratings of general health and psychological distress appear to be stronger than the effects of sexual aggression on these outcomes.

### 4.3. Hypotheses 4 (a, b, c): Joint Effects of General and Sexual Aggression on Outcomes

Finally, we performed a series of moderated regression analyses to examine the joint effects of general and sexual aggression on women’s outcomes—i.e., Hypothesis 4a, *additive effect*, Hypothesis 4b, *adaptive effect*, and Hypothesis 4c, *amplified effect*. For these analyses, we followed procedures described by R. M. Baron and Kenny (1986) and entered demographic variables in the first step, followed by general aggression and sexual aggression scores in the third step, and the interaction term in the final step for each of the outcomes we examined across all four groups of reservists included in the study. Interaction terms were mean-centered prior to analyses. Table 5 summarizes the results of these analyses.

As seen in Table 5, there was a significant interaction between general and sexual aggression at work, coworker, and leadership satisfaction and psychological stress among Traditional Reservists (TR), with effect sizes ranging from 0.02 to 0.23. There was also a significant interaction between general and sexual aggression on work and leadership satisfaction and psychological stress, with effect sizes ranging from 0.03 to 0.26 among reservists with prior activation history (RPAH). Finally, there was a significant interaction between general and sexual aggression on work and coworker satisfaction, with effect sizes ranging from 0.05 to 0.26 among reservists with recent activation history (RRAH). We followed procedures described by Aiken and West (1991) to construct plots for each of the significant interactions we found.

**Figure 1:**
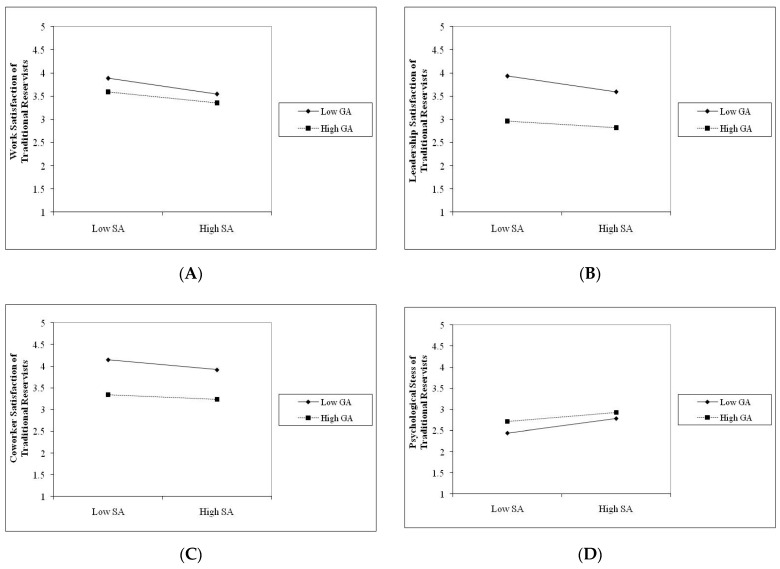
Joint effects of general and sexual aggression on satisfaction with work (**A**), coworkers (**B**), leaders (**C**) and psychological distress (**D**) among Traditional Reservists.

**Figure 2:**
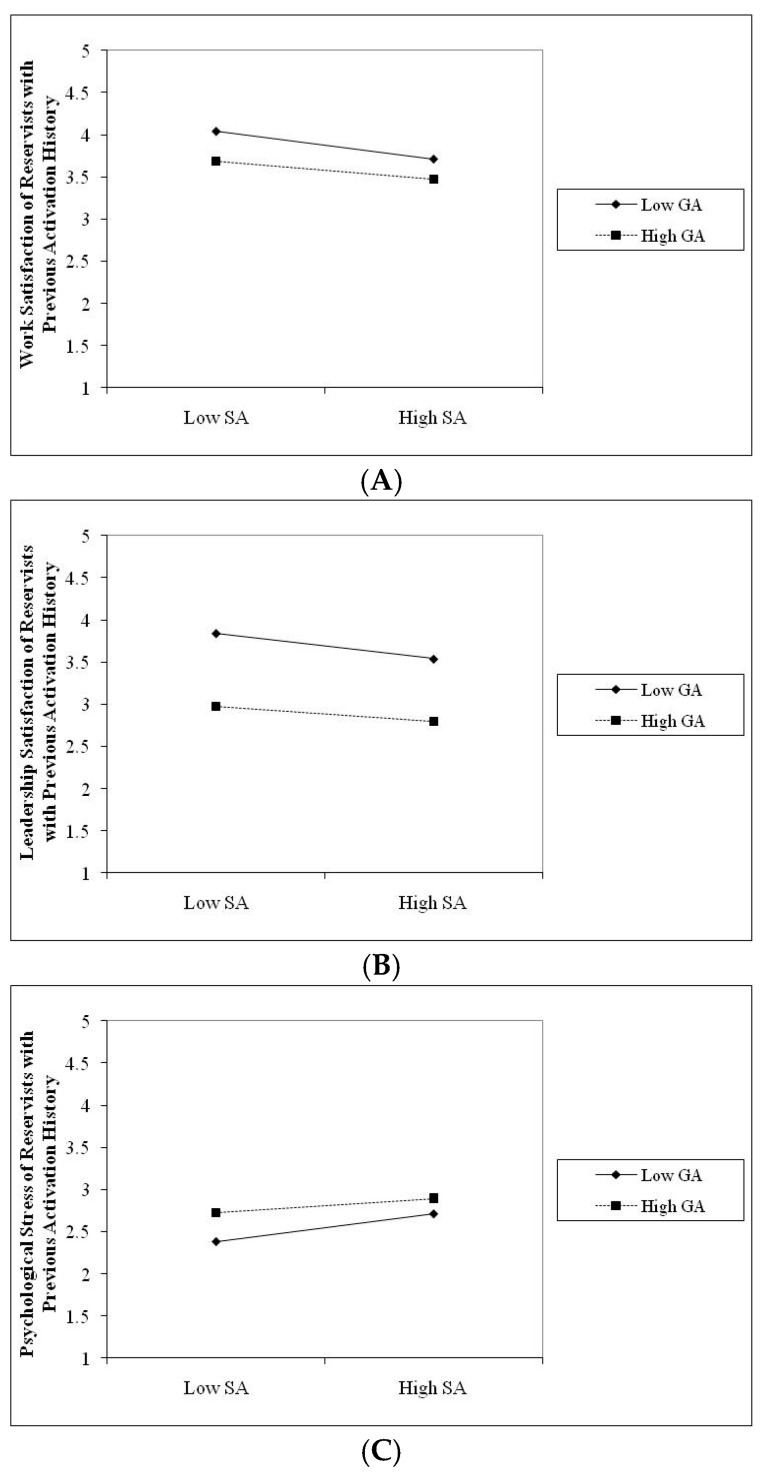
Joint effects of general and sexual aggression on satisfaction with work (**A**), leaders (**B**) and psychological distress (**C**) among reservists with previous activation history.

**Figure 3:**
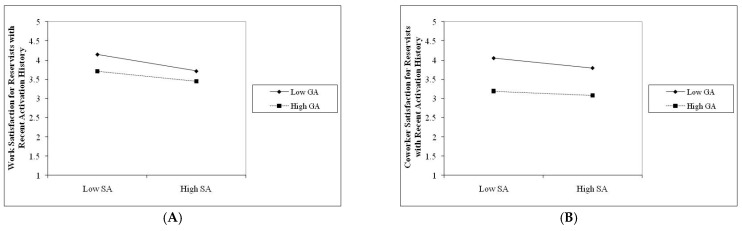
Joint effects of general and sexual aggression on satisfaction with work (**A**) and coworkers (**B**) among reservists with recent activation history.

As shown in Figure 1, Figure 2 and Figure 3, when the experience of general aggression was high, whether one experiences sexual aggression does not generally change the observed relationship between general aggression and satisfaction with work, coworkers and leaders. The pattern of findings is consistent for Traditional Reservists (TR—see Figure 1 Panel A, B and C), reservists with previous activation history (RPAH—see Figure 2 Panel A and B), and reservists with recent activation history (RRAH—see Figure 3 Panel A and B). These findings are most consistent with an adaptive interpretation (i.e., Hypothesis 4b). Recall that an adaptive effect implies that while general and sexual aggression may independently influence women’s satisfaction with work, coworkers, and leaders, their combination would not be significantly worse on women’s outcomes than the effects of experiencing one of these forms of aggression in isolation.

A slightly different pattern was observed with regard to the interaction involving general and sexual aggression on women’s psychological distress. As can be seen in Figure 1 and Figure 2, when the experience of general aggression was high, experiencing sexual aggression appears to change the observed relationship between general aggression and psychological distress—i.e., it increases levels of psychological distress. The pattern of findings is consistent for Traditional Reservists (TR—see Figure 1 Panel D) and for reservists with previous activation history (RPAH—see Figure 2 Panel C). While these findings appear to be most consistent with an amplification interpretation (i.e., Hypothesis 4c), we caution that, given the large sample sizes used in our analyses and the small effects observed, these findings, though statistically significant, may not be practically significant to be meaningful or consequential. Accordingly, we believe an adaptive interpretation (i.e., Hypothesis 4b) is most consistent with this pattern of findings. Thus, taken together, our results provide the strongest support for the adaptive hypotheses, since an adaptive effect is evident when the experience of both general and sexual aggression in combination does not predict significant incremental variance in women’s outcomes when compared to a single type of aggression alone.

## 5. Discussion

We examined the effects of general and sexual aggression on women’s jobs, health and psychological outcomes with data from women in the reserve component of the U.S. Armed Forces. Based on past theory and empirical research, we predicted that general and sexual aggression would negatively influence women’s jobs, health and psychological outcomes; and that the effects of general aggression on women’s jobs, health and psychological outcomes would be stronger than the effects of sexual aggression on women’s outcomes. We also evaluated competing hypotheses involving the joint effects of general and sexual aggression on women’s job, health and psychological outcomes—namely, that aggressive behaviors may combine in an additive, adaptive and amplified manner to influence women’s outcomes.

Consistent with our predictions, we found that both general and sexual aggression had the predicted negative effect on women’s jobs, health, and psychological outcomes. Moreover, the effects of general aggression were stronger than the effects of sexual aggression on women’s outcomes. Finally, we found that the joint effects of general and sexual aggression on women’s outcomes appear to be adaptive in nature rather than additive or amplified. It is worth noting that the pattern of findings was consistent across women reservists with varying activation and deployment histories. We discuss theoretical, methodological, and practical implications of these findings in the sections below.

### The Effects of General and Sexual Aggression on Women’s Outcomes

Correlational and regression analyses indicate that general and sexual aggression had a consistent pattern of negative effects on women’s jobs, health, and psychological outcomes (see Table 1, Table 2 and Table 3). Women who reported experiencing general or sexual aggression were more likely to be less satisfied with their work, coworkers, and leaders, and reported lower levels of organizational commitment, increased levels of psychological distress, and worse physical health. These findings are consistent with previous research documenting the negative effects of general aggression on women’s outcomes (e.g., Bowling & Beehr, 2006; Glomb, 2002; LeBlanc & Kelloway, 2002; Nielsen et al., 2008) and the negative effects of sexual aggression on women’s outcomes (e.g., Magley et al., 1999; Krieger et al., 2005; Willness et al., 2007). Interestingly, the pattern of findings for general and sexual aggression and women’s outcomes was remarkably consistent in both direction and magnitude, even though women had varying activation and deployment histories.

Results of hierarchical regression analyses evaluating the effects of general and sexual aggression on women’s outcomes suggest that after controlling for the effects of general aggression, many of the relationships among sexual aggression and women’s outcomes were reduced and, in some cases, were not statistically significant (see Table 4). These findings are consistent with findings reported in meta-analyses of this literature showing that the effects of general aggression tend to be stronger than the effects of sexual aggression on women’s outcomes (Hershcovis & Barling, 2010; Lapierre et al., 2005). Our results raise interesting questions as to why general aggression may have a stronger impact on women’s outcomes than sexual aggression. One possible explanation for this finding may be related to the fact that there are legal distinctions between general and sexual aggression. For example, sexual aggression is legally sanctioned, and most organizations have clear policies prohibiting these types of behavior. Indeed, while Duffy (2009) has noted that federal law prohibits aggression that is based on an individual’s protected class status (e.g., ethnicity, sex, religion), it offers no protections for general forms of aggressive behaviors. Thus, one reason why general aggression may be more damaging than sexual aggression may be related to the fact that organizational policies and procedures designed to curtail general forms of aggression are less developed than policies and procedures involving sexual forms of aggression. This finding is particularly important given that research has shown that organizational tolerance for sexual aggression influences women’s outcomes, independently of their individual experiences (Williams et al., 1999). Thus, it is possible that a lack of organizational support can lead to continued victimization and further damage an individual’s well-being and job-related attitudes (Cortina & Magley, 2003). It is also possible that general aggression may be more damaging than sexual aggression because of differences in how the behaviors are perceived. Hershcovis and Barling (2010) found that people are more likely to perceive general aggression as a personal attack, whereas sexual aggression may be more likely to be attributed to the perpetrator’s attitudes toward gender. Accordingly, this suggests that individuals may be more likely to attribute blame to the perpetrator for sexual forms of aggression, whereas they may be more likely to attribute blame to themselves for general forms of aggression (Hershcovis & Barling, 2010). However, we note that this interpretation is inconsistent with research, showing that targets of sexual aggression are more likely to blame themselves for the incident (K. M. Edwards et al., 2011; Gutek & Koss, 1993) and, as a result, may experience more guilt and shame and greater psychological distress (Bowling & Beehr, 2006; Drew & Chevroulet, 2024; Johansson et al., 2024). Clearly, additional research is needed to examine how attributions of blame influence the experience, perceptions, and outcomes of both general and sexual aggression.

In keeping with both theory (i.e., Helson, 1964; Hockey, 1997; Holmes & Rahe, 1967; Rahe & Arthur, 1978) and past research on the impact of multiple forms of workplace aggression (i.e., Raver & Nishii, 2010; MacIntosh et al., 2015), we evaluated three competing hypotheses regarding the joint effects of general and sexual aggression (i.e., additive, adaptive, and amplified effect). Both general and sexual aggression predicted significant proportions of incremental variance in job, health, and psychological outcomes; the predictive validity of sexual aggression on these outcomes was reduced once the effects of general aggression were taken into account (see Table 5). As shown in Figure 1, Figure 2 and Figure 3, under conditions of high general aggression, the experience of sexual aggression did not have a substantial impact on outcomes (see Figure 1, Figure 2 and Figure 3). Our results are consistent with those reported by Raver and Nishii (2010), who found that experiencing multiple forms of aggression did not have a significant additive impact on employees’ job outcomes beyond experiencing one form of aggression. Our findings are also consistent with adaptation-level theory (Helson, 1964), which suggests that when a person experiences general aggression, it may serve to normalize expectations regarding other forms of aggression at work (e.g., sexual aggression) and develop adaptive behavioral responses. In our study, women reporting higher levels of general aggression did not exhibit substantially stronger associations between sexual aggression and outcomes. This pattern is descriptively consistent with an adaptive rather than an additive or amplified response. Thus, adaptation-level theory may provide a useful framework for generating and testing hypotheses regarding how repeated or overlapping forms of aggression may shape perceptions and appraisals of these experiences within work organizations.

Importantly, we found that general and sexual aggression had a consistent pattern of negative effects on job, health, and psychological outcomes regardless of activation or deployment status. Our findings were consistent with past research demonstrating the negative impact of sexual aggression on part-time and full-time employees (e.g., Walsh & Hitlan, 2007; Herrmann et al., 2022), and the negative impact of general aggression on part-time and full-time employees (e.g., Dupré et al., 2006; Chambel et al., 2017). Thus, it appears that even infrequent exposure to aggressive behavior within the work environment can bring about the same negative effects as being exposed to these same behaviors on a more frequent basis.

As with all research, there were some limitations with this study. Most noteworthy is the cross-sectional nature of our data. Because participants were only surveyed once, we only have a snapshot of their experiences and perceptions. However, the results were consistent with past research on the impact of workplace aggression on job, health and psychological outcomes (e.g., Chan et al., 2008; Pacheco et al., 2021). Although it may be plausible that the relationships observed in these data may manifest in similar ways over time, longitudinal research is warranted to further validate these findings. One major advantage of this study, compared to other research on the impact of workplace aggression, is that participants were drawn through random stratified sampling, yielding a large sample that was representative of women reservists in the U.S. military. While reservists are inherently different from their active-duty counterparts, they share in common a military culture that makes it more likely that our findings may generalize to other military personnel as well as civilian employees. In fact, a recent meta-analysis examined the differential impact of sexual aggression on civilians as opposed to military personnel and found no significant differences in the observed association among sexual harassment experience and job, health and psychological outcomes (Willness et al., 2007).

It is worth noting that the statistical significance of results does not always imply practical significance, especially with large samples. However, the analytical strategy pursued sought to minimize the statistical impact of large sample sizes by breaking the entire sample (*n* = 13,541) into distinct sub-samples based on employment characteristics (e.g., activation and deployment experiences), thus lowering the sample size and allowing for the replication of results across four meaningfully distinct groups based on different levels of exposures to individuals (i.e., activation history) and to varying types of work environments (i.e., deployment history). We also recognize that a sizeable proportion of the variance in outcomes was not accounted for by our measures and subsequent model. Accordingly, future studies should examine the predictive validity of multiple forms of workplace aggression on outcomes after controlling for individual differences as well as other types of job stressors. Finally, it is important to acknowledge that the data used in these analyses is dated and may represent a limitation in the present study. However, this study aimed to examine the structural relationships between multiple forms of workplace aggression and employee outcomes that were derived from theory and past research. That said, future research with newer data could examine whether the observed pattern of effects is replicable in more contemporary civilian and military contexts. Such research would allow for the examination of the stability or variability of processes linking workplace aggression to job, health, and psychological outcomes over time.

## 6. Conclusions

In conclusion, the study demonstrated that both general and sexual aggression negatively affect women’s job satisfaction, health, and psychological well-being. The effects of general aggression were consistently stronger than those of sexual aggression across outcomes and groups, suggesting that general forms of aggressive behaviors may be particularly harmful in environments where organizational policies are less developed or not strongly enforced. Moreover, the joint effects of experiencing general and sexually aggressive behaviors at work appear to have an adaptive rather than additive or amplified quality. Thus, indicating that exposure to one form of aggression may reduce the impact of exposure to other forms of aggression on job, health, and psychological outcomes. These findings underscore the need for organizations to address all forms of workplace aggression through comprehensive policies and prevention strategies.

## Figures and Tables

**Table 1:** Zero-order correlation coefficients among study variables for Traditional Reservists (*n* = 9000) and for Reservists with Previous Activation History (*n* = 3153).

	M	SD	1	2	3	4	5	6	7	8	M	SD
1. Coworker Satisfaction	3.49	0.83	---	0.50	0.36	0.45	0.10	−0.16	−0.25	−0.45	3.65	0.79
2. Leadership Satisfaction	3.02	0.95	0.53	---	0.34	0.48	0.09	−0.14	−0.27	−0.48	3.26	0.89
3. Work Satisfaction	3.58	0.93	0.35	0.35	---	0.57	0.11	−0.19	−0.16	−0.16	3.65	0.96
4. Affective Commitment	3.29	1.08	0.46	0.52	0.51	---	0.14	−0.17	−0.19	−0.30	3.45	1.02
5. General Health	2.35	0.56	0.12	0.14	0.14	0.14	---	−0.37	−0.11	−0.13	3.38	0.55
6. Perceived Stress	2.31	0.74	−0.20	−0.17	−0.19	−0.18	−0.40	---	0.19	0.18	2.30	0.72
7. Sexual Aggression	1.31	0.51	−0.36	−0.32	−0.21	−0.26	−0.14	0.22	---	0.44	1.18	0.38
8. General Aggression	1.93	0.99	−0.50	−0.50	−0.23	−0.36	−0.14	0.26	0.50	---	1.64	0.82

Note: All coefficients are statistically significant, *p* < 0.01. Coefficients for Traditional Reservists appear above the diagonal and for Reservists with Previous Activation History appear below the diagonal.

**Table 2:** Zero-order correlation coefficients Among Study Variables for Reservists with Recent Activation History (*n* = 900) and for Reservists with Recent Activation and Deployment History (*n* = 638).

	M	SD	1	2	3	4	5	6	7	8	M	SD
1. Coworker Satisfaction	3.31	0.90	---	0.46	0.37	0.45	0.16	−0.27	−0.31	−0.50	3.48	0.87
2. Leadership Satisfaction	2.83	0.91	0.48	---	0.35	0.45	0.16	−0.28	−0.36	−0.50	3.06	0.92
3. Work Satisfaction	3.51	1.01	0.38	0.35	---	0.44	0.15	−0.30	−0.25	−0.26	3.79	0.91
4. Affective Commitment	3.14	1.09	0.45	0.41	0.41	---	0.16	−0.26	−0.27	−0.34	3.48	1.02
5. General Health	3.31	0.56	0.15	0.10 ^ns^	0.10 ^ns^	0.13	---	−0.36	−0.18	−0.21	3.36	0.56
6. Perceived Stress	2.56	0.79	−0.31	−0.27	−0.29	−0.25	−0.37	---	0.28	0.35	2.33	0.70
7. Sexual Aggression	1.42	0.54	−0.30	−0.36	−0.27	−0.26	−0.15	0.29	---	0.53	1.31	0.50
8. General Aggression	2.21	1.01	−0.54	−0.48	−0.28	−0.31	−0.22	0.39	0.55	---	2.02	0.98

Note: All coefficients are statistically significant, *p* < 0.01, unless noted otherwise (^ns^ = not significant). Coefficients for Reservists with Recent Activation History appear above the diagonal, and Reservists with Recent Activation and Deployment History appear below the diagonal.

**Table 3:** Summary of regression analyses examining the unique effects of general and sexual aggression on women’s outcomes.

		Work Sat.			Coworker Sat.			Leadership Sat.			Commitment			General Health			Psych. Stress		
		β	ΔF	ΔR^2^	β	ΔF	ΔR^2^	β	ΔF	ΔR^2^	β	ΔF	ΔR^2^	β	ΔF	ΔR^2^	β	ΔF	ΔR^2^
TR	Step I		3	0.00 ^ns^		18	0.00		53	0.01		49	0.01		89	0.02		136	0.03
	Min.	−0.02 ^ns^			−0.06			−0.10			−0.10			−0.02 ^ns^			0.04		
	Ed.	0.01 ^ns^			−0.02			−0.04			0.02 ^ns^			0.14			−0.17		
	Step II		164	0.04		1158	0.20		1325	0.23		444	0.09		87	0.02		234	0.05
	SA	−0.11			−0.06			−0.08			−0.08			−0.06			0.13		
	GA	−0.11			−0.42			−0.44			−0.26			−0.10			0.13		
RPAH	Step I		8	0.01		15	0.01		21	0.01		49	0.03		27	0.02		55	0.03
	Min.	−0.07			−0.09			−0.12			−0.17			−0.05			0.05		
	Ed.	−0.01 ^ns^			0.05			−0.00 ^ns^			0.00 ^ns^			0.12			−0.17		
	Step II		109	0.06		555	0.26		535	0.25		241	0.13		41	0.03		121	0.07
	SA	−0.13			−0.14			−0.10			−0.10			−0.10			0.12		
	GA	−0.17			−0.43			−0.45			−0.30			−0.09			0.18		
RRAH	Step I		2	0.00 ^ns^		4	0.01 ^ns^		10	0.01		15	0.02		17	0.02		20	0.03
	Min.	−0.03 ^ns^			−0.04 ^ns^			−0.10			−0.11			−0.01 ^ns^			0.02 ^ns^		
	Ed.	0.04 ^ns^			0.06 ^ns^			0.06 ^ns^			0.08			0.15			−0.16		
	Step II		68	0.08		259	0.25		271	0.26		107	0.12		39	0.05		111	0.12
	SA	−0.15			−0.06 ^ns^			−0.13			−0.13			−0.08			0.12		
	GA	−0.18			−0.47			−0.43			−0.27			−0.17			0.27		
RRADH	Step I		2	0.01 ^ns^		1	0.00 ^ns^		5	0.02		7	0.02		7	0.02		10	0.03
	Min.	−0.05 ^ns^			−0.04 ^ns^			−0.09 ^ns^			−0.11			0.01 ^ns^			0.01 ^ns^		
	Ed.	0.05 ^ns^			0.06 ^ns^			0.08 ^ns^			0.09 ^ns^			0.14			−0.17		
	Step II		32	0.09		128	0.29		99	0.24		38	0.11		15	0.05		54	0.14
	SA	−0.16			−0.01 ^ns^			−0.14			−0.13			−0.02 ^ns^			0.10 ^ns^		
	GA	−0.19			−0.54			−0.40			−0.24			−0.20			0.32		

Note: All coefficients are statistically significant, *p* < 0.01, unless noted otherwise (^ns^ = not significant). TR = Traditional Reservist (*n* = 8896). RPAH = Reservists with Previous Activation History (*n* = 3109). RRAH = Reservists with Recent Activation History (*n* = 1320). RRADH = Reservists with Recent Activation and Deployment History (*n* = 629). Min = Minority. Ed. = Education. GA = General Aggression. SA = Sexual Aggression.

**Table 4:** Summary of regression analyses examining the differential effects of general and sexual aggression on women’s outcomes.

		Work Sat.			Coworker Sat.			Leadership Sat.			Commitment			General Health			Psych. Stress		
		Β	ΔF	ΔR^2^	β	ΔF	ΔR^2^	Β	ΔF	ΔR^2^	β	ΔF	ΔR^2^	β	ΔF	ΔR^2^	β	ΔF	ΔR^2^
TR	Step I		3	0.00		18	0.00		53	0.01		49	0.01		89	0.02		136	0.03
	Min.	−0.02 ^ns^			−0.06			−0.10			−0.10			−0.02 ^ns^			0.04		
	Ed.	0.01 ^ns^			−0.02 ^ns^			−0.04			0.02 ^ns^			0.14			−0.17		
	Step II		234	0.03		598	0.06		707	0.07		336	0.04		99	0.01		337	0.04
	SA	−0.16			−0.25			−0.27			−0.19			−0.10			0.19		
	Step III		93	0.01		1612	0.14		1802	0.15		531	0.05		74	0.01		126	0.01
	GA	−0.11			−0.42			−0.44			−0.26			−0.10			0.13		
	Step I		3	0.00 ^ns^		18	0.00		53	0.01		49	0.01 ^ns^		89	0.02		136	0.03
	Min.	−0.02 ^ns^			−0.06			−0.10			−0.10			−0.02 ^ns^			0.04 ^ns^		
	Ed.	0.01 ^ns^			−0.02 ^ns^			−0.04			0.02 ^ns^			0.14			−0.17		
	Step II		236	0.03		2271	0.20		2582	0.22		838	0.08		147	0.02		328	0.03
	GA	−0.16			−0.45			−0.47			−0.29			−0.13			0.19		
	Step III		91	0.01		37	0.00		53	0.01		45	0.01		27	0.00		135	0.01
	SA	−0.11			−0.06			−0.08			−0.08			−0.06			0.13		
RPAH	Step I		8	0.01		15	0.01		21	0.01		49	0.03		27	0.02		55	0.03
	Min.	−0.07			−0.09			−0.12			−0.17			−0.05			0.05		
	Ed.	−0.01 ^ns^			0.05			−0.00 ^ns^			0.00 ^ns^			0.12			−0.17		
	Step II		146	0.04		450	0.12		360	0.10		218	0.06		63	0.02		153	0.05
	SA	−0.21			−0.35			−0.32			−0.25			−0.14			0.21		
	Step III		68	0.02		577	0.13		638	0.15		248	0.07		19	0.01		84	0.02
	GA	−0.17			−0.43			−0.45			−0.30			−0.09			0.18		
	Step I		8	0.01		15	0.01		21	0.01		49	0.03		27	0.02		55	0.03
	Min.	−0.07			−0.09			−0.12			−0.17			−0.05			0.05		
	Ed.	−0.01 ^ns^			0.05			−0.00 ^ns^			0.00 ^ns^			0.12			−0.17		
	Step II		173	0.05		1024	0.24		1032	0.25		449	0.12		59	0.02		199	0.06
	GA	−0.23			−0.50			−0.50			−0.35			−0.14			0.24		
	Step III		42	0.01		64	0.02		30	0.01		30	0.01		23	0.01		40	0.01
	SA	−0.13			−0.14			−0.10			−0.10			−0.10			0.12		
RRAH	Step I		0.21	0.00 ^ns^		1	0.01		0.20	0.00 ^ns^		6	0.04		6	0.04		2	0.01 ^ns^
	Min.	0.01 ^ns^			−0.07 ^ns^			−0.02 ^ns^			−0.17			−0.15 ^ns^			0.05 ^ns^		
	Ed.	0.04 ^ns^			0.02 ^ns^			0.03 ^ns^			0.09 ^ns^			0.12 ^ns^			−0.11 ^ns^		
	Step II		2	0.01 ^ns^		37	0.11		64	0.18		17	0.05		18	0.06		32	0.10
	SA	−0.09 ^ns^			−0.33			−0.42			−0.23			−0.24			0.31		
	Step III		8	0.03		24	0.07		56	0.13		36	0.10		10	0.03		11	0.03
	GA	−0.20			−0.32			−0.45			−0.39			−0.21			0.23		
	Step I		2	0.00 ^ns^		4	0.01 ^ns^		10	0.01		15	0.02		17	0.02			0.03
	Min.	−0.03 ^ns^			−0.04 ^ns^			−0.10			−0.11			−0.01 ^ns^			0.02 ^ns^		
	Ed.	0.04 ^ns^			0.06 ^ns^			0.06 ^ns^			0.08			0.15			−0.16		
	Step II		108	0.07		512	0.25		505	0.24		192	0.11		70	0.04			0.11
	GA	−0.26			−0.50			−0.50			−0.33			−0.21			0.34		
	Step III		26	0.02		5	0.00 ^ns^		27	0.01		20	0.01		8	0.01			0.01
	SA	−0.15			−0.06 ^ns^			−0.13			−0.13			−0.08			0.12		
RRADH	Step I		2	0.01 ^ns^		1	0.00 ^ns^		5	0.02		7	0.02		7	0.02		10	0.03
	Min.	−0.05 ^ns^			−0.04 ^ns^			−0.09 ^ns^			−0.11			0.01 ^ns^			0.01 ^ns^		
	Ed.	0.05 ^ns^			0.06 ^ns^			0.08 ^ns^			0.09 ^ns^			0.14			−0.17		
	Step II		46	0.07		62	0.09		92	0.13		44	0.07		11	0.02		52	0.07
	SA	−0.26			−0.30			−0.36			−0.26			−0.13			0.27		
	Step III		17	0.03		177	0.20		93	0.11		29	0.04		19	0.03		53	0.07
	GA	−0.19			−0.54			−0.40			−0.24			−0.20			0.32		
	Step I		2	0.01 ^ns^		1	0.00 ^ns^		5	0.02		7	0.02		7	0.02		10	0.03
	Min.	−0.05 ^ns^			−0.04 ^ns^			−0.09 ^ns^			−0.11			0.01 ^ns^			0.01 ^ns^		
	Ed.	0.05 ^ns^			0.06 ^ns^			0.08 ^ns^			0.09 ^ns^			0.14			−0.17		
	Step II		51	0.08		256	0.29		183	0.22		67	0.09		30	0.05		103	0.14
	GA	−0.28			−0.54			−0.48			−0.31			−0.21			0.37		
	Step III		13	0.02		0.09	0.00 ^ns^		12	0.01		8	0.01		0.26	0.00 ^ns^		5	0.01 ^ns^
	SA	−0.16			0.01 ^ns^			−0.14			−0.13			−0.02 ^ns^			0.10 ^ns^		

Note: All coefficients are statistically significant, *p* < 0.01, unless noted otherwise (^ns^ = not significant). TR = Traditional Reservist (*n* = 8896). RPAH = Reservists with Previous Activation History (*n* = 3109). RRAH = Reservists with Recent Activation History (*n* = 1520). RRADH = Reservists with Recent Activation and Deployment History (*n* = 629). Min = Minority. Ed. = Education. GA = General Aggression. SA = Sexual Aggression.

**Table 5:** Summary of moderated regression analyses for general and sexual aggression on women’s outcomes.

		Work Sat.			Coworker Sat.			Leadership Sat.			Commitment			General Health			Psych. Stress		
		β	ΔF	ΔR^2^	β	ΔF	ΔR^2^	β	ΔF	ΔR^2^	β	ΔF	ΔR^2^	β	ΔF	ΔR^2^	β	ΔF	ΔR^2^
TR	Step I		3	0.00 ^ns^		18	0.00		53	0.01		49	0.01		89	0.02		136	0.03
	Min.	−0.02 ^ns^			−0.06			−0.10			−0.10			−0.02 ^ns^			0.04		
	Ed.	0.01 ^ns^			−0.02			−0.04			0.02 ^ns^			0.14			−0.17		
	Step II		164	0.04		1158	0.20		1325	0.23		444	0.09		87	0.02		234	0.05
	SA	−0.11			−0.06			−0.08			−0.08			−0.06			0.13		
	GA	−0.11			−0.42			−0.44			−0.26			−0.10			0.13		
	Step III		8	0.00		21	0.00		43	0.00		4	0.00 ^ns^		3	0.00 ^ns^		25	0.00
	SA × GA	0.04			0.05			0.08			0.03 ^ns^			0.02 ^ns^			−0.06		
RPAH	Step I		8	0.01		15	0.01		21	0.01		49	0.03		27	0.02		55	0.03
	Min.	−0.07			−0.09			−0.12			−0.17			−0.05			0.05		
	Ed.	−0.01 ^ns^			0.05			−0.00 ^ns^			0.00 ^ns^			0.12			−0.17		
	Step II		109	0.06		555	0.26		535	0.25		241	0.13		41	0.03		121	0.07
	SA	−0.13			−0.14			−0.10			−0.10			−0.10			0.12		
	GA	−0.17			−0.43			−0.45			−0.30			−0.09			0.18		
	Step III		7	0.00		0.35	0.00 ^ns^		10	0.00		1	0.00 ^ns^		4	0.00 ^ns^		22	0.01
	SA × GA	0.07			0.01 ^ns^			0.07			0.03 ^ns^			0.05 ^ns^			−0.12		
RRAH	Step I		2	0.00 ^ns^		4	0.01 ^ns^		10	0.01		15	0.02		17	0.02		20	0.03
	Min.	−0.03 ^ns^			−0.04 ^ns^			−0.10			−0.11			−0.01 ^ns^			0.02 ^ns^		
	Ed.	0.04 ^ns^			0.06 ^ns^			0.06 ^ns^			0.08			0.15			−0.16		
	Step II		68	0.08		259	0.25		271	0.26		107	0.12		39	0.05		111	0.12
	SA	−0.15			−0.06 ^ns^			−0.13			−0.13			−0.08			0.12		
	GA	−0.18			−0.47			−0.43			−0.27			−0.17			0.27		
	Step III		8	0.01		8	0.00 ^ns^		3	0.00 ^ns^		1	0.00 ^ns^		0.06	0.00 ^ns^			0.00 ^ns^
	SA × GA	0.11			0.10			0.06 ^ns^			0.04 ^ns^			0.01 ^ns^			−0.08 ^ns^	4	
RRADH	Step I		2	0.01 ^ns^		1	0.00 ^ns^		5	0.02		7	0.02		7	0.02		10	0.03
	Min.	−0.05 ^ns^			−0.04 ^ns^			−0.09 ^ns^			−0.11			0.01 ^ns^			0.01 ^ns^		
	Ed.	0.05 ^ns^			0.06 ^ns^			0.08 ^ns^			0.09 ^ns^			0.14			−0.17		
	Step II		32	0.09		128	0.29		99	0.24		38	0.11		15	0.05		54	0.14
	SA	−0.16			−0.01 ^ns^			−0.14			−0.13			−0.02 ^ns^			0.10 ^ns^		
	GA	−0.19			−0.54			−0.40			−0.24			−0.20			0.32		
	Step III		0.27	0.00 ^ns^		0.01	0.00 ^ns^		2	0.00 ^ns^		1	0.00 ^ns^		0.00	0.00 ^ns^		1	0.00 ^ns^
	SA × GA	−0.03 ^ns^			0.01 ^ns^			0.09 ^ns^			0.06 ^ns^			0.00 ^ns^			−0.05 ^ns^		

Note: All coefficients are statistically significant, *p* < 0.01, unless noted otherwise (^ns^ = not significant). TR = Traditional Reservist (*n* = 8896). RPAH = Reservists with Previous Activation History (*n* = 3109). RRAH = Reservists with Recent Activation History (*n* = 1520). RRADH = Reservists with Recent Activation and Deployment History (*n* = 629). Min = Minority. Ed. = Education. GA = General Aggression. SA = Sexual Aggression.

## Data Availability

Data from the study may be obtained via a Freedom of Information (FOIA) request to the DoD Data Management Center.

## Notes

1 The Study Response Project is an academic, non-profit, web-based research referral system hosted by the School of Information Studies at Syracuse University. The project seeks to facilitate online data collection for researchers in the behavioral, social, and organizational sciences by maintaining a list of self-selected adult subscribers who sign up to participate in online research studies.

2 Study 1 had 226 university library employees; Study 2 had 586 internet respondents and 149 employed business students.
